# Examining the quality of record linkage process using nationwide Brazilian administrative databases to build a large birth cohort

**DOI:** 10.1186/s12911-020-01192-0

**Published:** 2020-07-25

**Authors:** Daniela Almeida, David Gorender, Maria Yury Ichihara, Samila Sena, Luan Menezes, George C. G. Barbosa, Rosimeire L. Fiaccone, Enny S. Paixão, Robespierre Pita, Mauricio L. Barreto

**Affiliations:** 1 Centre for Data and Knowledge Integration for Health (CIDACS), Fiocruz Bahia, Salvador, Brazil; 2 University of Arizona, Computer Science Department, Tucson, Arizona USA; 3 Department of Statistics, Federal University of Bahia (UFBA), Salvador, Brazil; 4 Epidemiology and Population Health, London School of Hygiene and Tropical Medicine, London, UK

## Abstract

**Background:**

Research using linked routine population-based data collected for non-research purposes has increased in recent years because they are a rich and detailed source of information. The objective of this study is to present an approach to prepare and link data from administrative sources in a middle-income country, to estimate its quality and to identify potential sources of bias by comparing linked and non-linked individuals.

**Methods:**

We linked two administrative datasets with data covering the period 2001 to 2015, using maternal attributes (name, age, date of birth, and municipally of residence) from Brazil: live birth information system and the 100 Million Brazilian Cohort (created using administrative records from over 114 million individuals whose families applied for social assistance via the Unified Register for Social Programmes) implementing an in house developed linkage tool CIDACS-RL. We then estimated the proportion of highly probably link and examined the characteristics of missed-matches to identify any potential source of bias.

**Results:**

A total of 27,699,891 live births were submited to linkage with maternal information recorded in the baseline of the 100 Million Brazilian Cohort dataset of those, 16,447,414 (59.4%) children were found registered in the 100 Million Brazilian Cohort dataset. The proportion of highly probably link ranged from 39.3% in 2001 to 82.1% in 2014. A substantial improvement in the linkage after the introduction of maternal date of birth attribute, in 2011, was observed. Our analyses indicated a slightly higher proportion of missing data among missed matches and a higher proportion of people living in an urban area and self-declared as Caucasian among linked pairs when compared with non-linked sets.

**Discussion:**

We demonstrated that CIDACS-RL is capable of performing high quality linkage even with a limited number of common attributes, using indexation as a blocking strategy in larg e routine databases from a middle-income country. However, residual records occurred more among people under worse living conditions. The results presented in this study reinforce the need of evaluating linkage quality and when necessary to take linkage error into account for the analyses of any generated dataset.

## Background

Research using routine population-based data collected for social, financial, and clinical purposes has increased in recent years because they are a rich and detailed source of information available at a relatively low cost [1]. Record linkage (process used to bring together information recorded in different sources about the same individual) [2] of multiples databases can further enhance the ability to answer scientific questions. Research using linked data has become common, especially in high-income countries [3, 4]; however, for low and middle-income countries, record linkage methods have only been developed more recently [5]. On maternal and infant health, linked data are a valuable source of information since it can increase the availability of information on maternal health, social, and economic trajectories before and during pregnancy [3].

Record linkage can be conducted using two main methods: deterministic and probabilistic. Deterministic linkage usually uses a unique identifier or a set of several attributes present in all the databases to be linked [6]. Probabilistic record linkage solutions are suitable when there is not a shared key to identify unequivocally an individual across disparate data sources [7, 8]. This situation is frequent in different countries, in particular in low and middle-income ones. To perform this procedure, we have to submit the most reliable and discriminative variables present in both databases to calculate similarity scores representing the likelihood that two records belong to the same person. The similarity score is used to classify records as links, non-links, and uncertain links based on one or more thresholds. The choice of threshold needs to balance the risk of “false-matches” (records from different individuals that are mistakenly linked) and “missed-matches” (records from the same individual that fail to link) [9].

Some extensions of linkage error in administrative data are expected and inevitable due to the imperfect and transient nature of the attributes. However, even a small amount of linkage error can lead to biased results, diluting real association, or creating spurious ones [10]. Measures of sensitivity, specificity, positive and negative predictive values are commonly used to estimate the linkage accuracy. Nevertheless, results of linkage accuracy by itself might not indicate in which extend the results of analyses using the linked data could be biased, because even small percentage of linkage error when does not occur randomly throughout the sample could introduce biased results. For example, if particular subgroups of records are less or more likely to link. Therefore, it is essential to combine these measures with alternative methods to evaluate linkage quality [11].

We aimed to use Brazilian nationwide administrative databases to build CIDACS (Centre for Data and Knowledge Integration for Health) birth cohort that will be originated by the link between the live births dataset and the baseline of the 100 Million Brazilian Cohort (created using administrative records from over 114 million individuals whose families applied for social assistance via the National Register for Social Programmes). The design of CIDACS Birth Cohort follows a life course perspective, using routinely collected data from Brazil. The use of linked high-quality administrative datasets provides a unique opportunity to examine factors that might result in long-term and rare child and maternal outcomes over time, with the additional advantage of using large samples, little loss to follow-up, high level of external validity and a great deal of applicability for policymaking [11–13].

We expected an overlap between the baseline of the 100 Million Brazilian Cohort and the live birth databases. In this scenario, we were able to measure the linkage error. This study presents an approach to prepare and link data from administrative sources in a middle-income country, estimating the proportion of births for which you were able to identify a link based on a specified threshold and identifying potential sources of bias by comparing link and no-links.

## Methods

In this section, we describe the methods we used to integrate two major nationwide databases, the Live Birth Information System (SINASC) and the baseline of the 100 Million Brazilian Cohort from 2001 to 2015.

### Datasets

1. SINASC (Sistema de Informação Sobre Nascidos Vivos/ Live Birth Information System)

The Brazilian Ministry of Health defines live births as the complete expulsion or extraction from the body of the pregnant woman of a product of conception, independent of the duration of pregnancy, who, after the separation, breathes or shows any other signs of life, such as heartbeat, umbilical cord pulsation, or definite movement of voluntary muscles, whether or not the cord is cut and whether or not the placenta is attached. SINASC records live births in Brazil, and this system is updated using the registration of live birth. It is a compulsory document, completed by a health professional who assisted the delivery. This form is divided into eight blocks. I -characteristics of the newborn; II- identification of the place of birth; III- characteristics of the mother; IV- identification of the father; V- characteristics of pregnancy and delivery; VI- characteristics of congenital anomalies: this block should be filled in when congenital anomalies are identified at birth using the ICD-10 code. VII- identification of the professional completing the notification. VIII- registry office identification [14]. Between 2001 to 2015 this system recorded 44.485.274 births.

Data completeness is very high, with 97% of Brazilian births registered [15], and most variables were >90% complete.

2) The baseline of the 100 Million Brazilian Cohort

The baseline of the 100 million Brazilian cohort was built using information from the application of families and their family members for social assistance programmes in Brazil through the registration with the Unified Register for Social Programs (CadÚnico). The CadÚnico is the main instrument used by the Brazilian government to assess the inclusion criteria of potential beneficiaries of social programs. To be enrolled in CadÚnico, one person in the family must provide information and required documents of all family members to an interviewer. This person must be at least 16 years old and, preferably, be a woman. The information available in the 100 million cohort is collected for each member of the family in a standardized form that includes individual (ie, sex, age, race or ethnicity, education, and work status) and familial (ie, familial income, household density, and housing characteristics) sociodemographic information. The information is renewed periodically as long as the person is a candidate to receive one of the several Brazilian government social protection programs [16]. The Centre for Data and Knowledge Integration for Health - CIDACS has the custody of several snapshots of CadÚnico. Each snapshot file refers to a year backup from 2001 to 2015. The efforts to build the 100 Million Brazilian Cohort were concentrated in three main steps. The first was the harmonization of variables from three different versions of CadÚnico. Second, the data cleaning to ensure the standardization of the categories. The third step aims to find the first appearance of each individual in the CadÚnico backup file.

Data completeness depend on the variable, but name and municipality of residence are available for all individual registered. Once registered, each family receive a unique code.

### The process of linking

#### Data pre-processing

During the data pre-processing phase, first, we searched automatically for invalid names (e.g., “unknown” or “newborn”), by comparing the recorded name with a standardized list of possible Brazilian names. All names considered invalid are submitted to a clerical review. In this review, the potential invalid terms are analysed to see if they are valid but were not recognized because they had typos, different spelling, or foreign name, among other reasons, or if in fact; they are invalid (such as RN from, unknown, ignored). And so, any term that deviates from what is known as “valid” is excluded. We removed punctuation, deleted consecutive spaces; middle initials, prefixes, and suffixes were maintained as recorded to retain the discriminatory power of the name variable.

#### Blocking/ Indexing

The complexity of the record linkage task is quadratic. We have to find the best match, on database B, for each record in database A, |A| X |B|. ‘To enable the record linkage is efficient when massive datasets are involve, we need to use methods capable of avoiding unnecessary comparisons, whilst keeping the accuracy. The total number of pairwise comparisons between SINASC and CadUnico would otherwise be prohibitively high 44,485,267 x 114,007,705=5,07166e15. To meet these challenges, we use the CIDACS-RL [16] (Centre for Data and Knowledge Integration for Health- record linkage); a novel record linkage tool developed to link big administrative datasets at the CIDACS (Centre for Data and Knowledge Integration for Health).

The CIDACS-RL applies the combination of indexing and searching algorithms implemented in Apache Lucene solution as the blocking strategy to reduce the number of comparisons during the linkage. The indexation strategy allows the CIDACS-RL to search the most similar records from the Indexed baseline of the 100 Million Brazilian Cohort for each record in SINASC and submit them to the pairwise comparisons step, instead of restricting the comparison group as an ordinary blocking step. This search was performed in two ways, (i) using the mothers’ name, municipality, and mothers date of birth records as attributes, from 2011 to 2015 (ii) using mothers name and municipality, from 2001-2010, because the mothers’ date of birth was not registered before 2011. This search strategy uses a mixture of exact, semi fuzzy and fuzzy queries to return the 1000 best candidates from the indexed baseline of the 100 Million Brazilian Cohort. The exact queries return only records with equal attributes in every querying, while the semi-fuzzy and fuzzy approaches permit more flexibility by retrieving candidates where one (semi-fuzzy) or more attributes differ (fuzzy). In cases where the name of the mother was not the same, the Damerau-Levenshtein distance is used as a string comparator to estimate the similarity between comparison pairs, and values above 0.5 are considered [17].

#### Pairwise Comparison

The most discriminant variables available on the live birth database to identify a child are a maternal name, maternal municipality, and maternal age. For those records from 2011 to 2015, the mothers’ date of birth attribute becomes available, and its filling increases gradually across the years. For 2001-2010, where the mothers date of birth is not available, we proceeded with the search using only two attributes (mothers name and municipality) then, we create a new variable by subtracting the date of birth of the child information recorded in SINASC from the date of birth of the mother recorded in baseline of the 100 Million Brazilian Cohort, and this value was compared with the age of the mother registered in SINASC, only the candidates with exacted same value were considered as possible candidates and submitted to the pairwise comparison step. This step was also executed for records from 2011 to 2015 with missing values in the mothers’ date of birth.

**Fig. 1:**
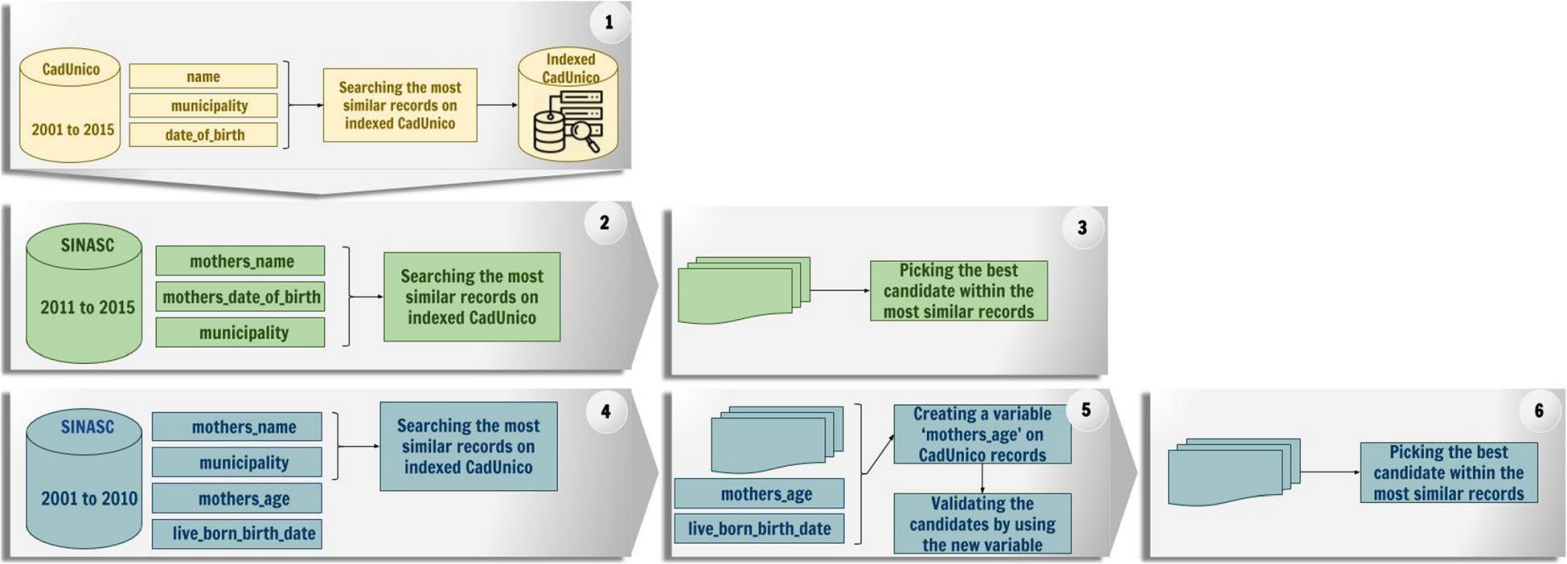
Flow chart data linkage tool

Figure 1 describes the two different approaches for each set of available variables. Then CIDACS-RL set weights according to the discriminatory power of the attributes ( name of the mother: 1 maternal age or date of birth: 1 state of birth: 0.008, municipality of birth: 0.16). At that moment, a combined scoring and query modules are used to perform the record linkage.

The similarities between names recorded in SINASC and the 1000 best candidates from the baseline of the 100 Million Brazilian Cohort were compared using the Jaro-Winkler string comparator [18]. The Jaro-Winkler string comparator [19] counts the number of common characters between two strings and the number of transpositions of these common characters, producing similarity values varying between 0 and 1 (perfectly similar). To compare the date attributes, we applied the Hamming distance [16], which measures the minimum number of substitutions required to change one string into the other. Then a linkage score is generated, and the function returns all pairs matched along with the score obtained.

#### Selection of the threshold

Candidate linking records were ordered by the scores achieved; only the comparison pair with the highest score is retained as a potential link. All remaining candidate records are discarded. If two people received the same candidate as a potential link, we retained only the ‘best candidate’ as a comparison pair. We removed this candidate as a possible match for all other comparison pair. Then a sample of 2000 pairs stratified in three categories of linkage score (high score – above 0.95, intermediate score – values between 0.90 and 0.95, and low score - below 0.90) is evaluated manually, and the records pairs are classified as likely true pairs or likely false pairs. Based on the training dataset of 2000, the receiver operating curve (ROC) is built to choose the best cut off point, and calculating the area under the curves (AUC), balancing between sensitivity and specificity values. Records were therefore classified as links or non-links based on a single threshold. The software R is used to generate accurate results.

#### Evaluation of the linkage error

Since we expected that all births registered in the baseline of the 100 Million Brazilian Cohort overlapped with the births existing at SINASC databases, we were able to identify the number of missed matches (record from the same mother-baby pair that failed to link) of the linkage. We then examined which characteristics were associated with missed matches. We examined race, sex, place of residence, sewage treatment, water supply, garbage collection.

The process described above identifies maternal links between the SINASC and the 100 million cohort dataset. After the mother is identified, we searched for the registry of the offspring in the 100 million cohorts using the child date of birth and sex.

## Results

A total of 27,699,891 live births were submitted to linkage with maternal information recorded in the baseline of the 100 Million Brazilian Cohort dataset from 2001 to 2015. Of those, 16,447,414 (59,4%) children were found registered in the 100 Million Brazilian Cohort dataset. However, the proportion of liked pairs were not similar over the years (Table 1). In general, the proportion of births for which you were able to identify a link based on a specified threshold of the linkage improved over the years. It ranged from 39.3% in 2001 to 82.1% in 2014. The greatest improvement was observed from 2010 to 2011 when the proportion of links increased by 10% (Table 1). The inclusion of the date of mother’s birth attribute provided a better discriminatory power when compared with maternal age, as indicated by the area under the ROC curve. For example, in 2011, the AUC in the records that included the maternal date of birth was 99.36%, which was higher than records that only included the maternal age AUC 95.59% in 2011, for example (Table 2).

**Table 1:** Number and percentage of linked records by year, Brazil, 2001-2015

Year	Total	Linked	
		N	%
**2001**	2,448,609	961,605	39.27
**2002**	2,319,071	1,175,223	50.68
**2003**	2,224,872	1,179,781	53.03
**2004**	2,165,661	1,144,809	52.86
**2005**	2,161,484	1,183,292	54.74
**2006**	2,050,534	1,271,179	61.99
**2007**	1,961,446	1,087,254	55.43
**2008**	1,936,675	1,077,781	55.65
**2009**	1,855,919	1,052,394	56.70
**2010**	1,778,515	1,067,417	60.02
2011*	1,765,211	1,249,492	70.78
**2012**	1,662,414	1,251,251	75.27
**2013**	1,505,476	1,227,162	81.51
**2014**	1,271,156	1,043,499	82.09
**2015**	592,848	475,275	80.17
**Total**	27,699,891	16,447,414	59.38

*from 2011 the maternal date of birth was available

**Table 2:** Metrics of accuracy - Linkage for mother

Year	Date of mother’s birth available							
	No				Yes			
	AUC (%)	Threshold	Specificity (%)	Sensitivity (%)	AUC (%)	Threshold	Specificity (%)	Sensitivity (%)
**2001**	99.18	0,929	96,4	95,5	---	---	---	---
**2002**	98.04	0,928	92,2	96,2	---	---	---	---
**2003**	98.94	0,9300	95,2	96,5	---	---	---	---
**2004**	99.31	0,954	98,4	94,6	---	---	---	---
**2005**	99.34	0,947	97,4	96,1	---	---	---	---
**2006**	93.94	0,915	81,6	96,4	---	---	---	---
**2007**	96.04	0,954	90,3	96,1	---	---	---	---
**2008**	95.74	0,955	88,7	97,7	---	---	---	---
**2009**	96.63	0,950	87,4	98,2	---	---	---	---
**2010**	98.50	0,944	93,5	98,6	---	---	---	---
**2011**	95.59	0,955	86,6	97,6	99.36	0,940	96,9	98,7
**2012**	96.79	0,925	88,1	97,4	98.58	0,941	96,1	94,1
**2013**	97.19	0,952	88,5	98,6	98.25	0,920	95,6	94,7
**2014**	96.70	0,953	86,7	97,9	98.20	0,913	93,1	95,5
**2015**	97.28	0,955	88,3	98,4	99.15	0,933	97,1	94,5

In general, missed-matches had a higher proportion of missing data in some living conditions variables such as water supply, sewage treatment, garbage collection, compared with linked pairs. According to the socio-demographic's characteristics, the linked group was more likely to live in an urban area and self-declared as Caucasian when compared with non-linked pairs (Table 3).

**Table 3:** Associations between the characteristics of the cohort and the accuracy of the linkage

Characteristics	2001				2014			
	Linked		Non-linked		Linked		Non-linked	
**Water supply**								
Missing	11610	0.78	11610	0.78	50098	4.80	14134	6.21
Public supply	982902	66.10	982902	66.10	735652	70.50	150925	66.29
Well	361618	24.32	361618	24.32	171268	16.41	41885	18.40
Other	130874	8.80	130874	8.80	86481	8.29	20713	9.10
**Sanitary sewage**								
Missing	8188	0.85	22896	1.54	138178	13.24	37445	16.45
Public collection	378673	39.38	616471	41.46	439186	42.09	83050	36.48
Septic tank	158983	16.53	207798	13.97	137642	13.19	31112	13.67
Rudimentary Pit	253761	26.39	371292	24.97	285395	27.35	65959	28.97
Ditch	143119	14.88	237890	16.00	35164	3.37	8143	3.58
Other	18881	1.96	30657	2.06	7934	0.76	1948	0.86
**Waste destination**								
Missing	5518	0.57	11616	0.78	50098	4.80	14134	6.21
Collected	698035	72.59	1035356	69.63	793622	76.05	162792	71.51
Burnt / Buried	173667	18.06	294548	19.81	174553	16.73	44385	19.50
Landfill	75287	7.83	127226	8.56	19263	1.85	4846	2.13
Other	9098	0.95	18258	1.23	5963	0.57	1500	0.66
**Education**								
Missing	33774	3.51	64030	4.31	42891	4.11	8903	8.30
Pre-school	149013	15.50	199390	13.41	14640	1.40	2946	1.29
Literacy	63098	6.56	84264	5.67	84	0.01	27	0.01
Elementary school	204054	21.22	455525	30.63	410	0.04	297	0.13
High school	956	0.10	2062	0.14	187	0.02	83	0.04
College education	52	0.01	108	0.01	0	0.00	2	0.00
Illiteracy	510658	53.10	681625	45.84	985287	94.42	215399	94.62
**Race/colour**								
Missing	21525	2.24	25275	1.70	1	0.00	0	0.00
Caucasian	312717	32.52	474201	31.89	339022	32.49	64997	28.55
Black	56836	5.91	78562	5.28	35608	3.41	7596	3.34
Asian	3465	0.36	4667	0.31	4932	0.47	1203	0.53
Brown	562957	58.54	890725	59.90	654706	62.74	151017	66.34
Indigenous	4105	0.43	13574	0.91	9230	0.88	2844	1.25
**Sex**								
Male	491672	51.13	763571	51.35	533728	51.15	114985	50.51
Female	469933	48.87	723433	48.65	509771	48.85	112672	49.49
**Zone**								
Missing	123	0.01	405	0.03	126	0.01	26	0.01
Urban	724567	75.35	1079682	72.61	808507	77.48	168299	73.93
Rural	236915	24.64	406917	27.36	234866	22.51	59332	26.06

2001- before maternal date of birth was available, 2014- after maternal date of birth was available

## Discussion

We have implemented the linkage tool CIDACS-RL [18] developed in house in a dataset with a known number of expected matches and consequently were able to quantify the proportion of births for which you were able to identify a link based on a specified threshold. We demonstrated that CIDACS-RL is capable of performing high quality linkage even with a limited number of common attributes, using indexation as a blocking strategy in a large routine dataset from a middle-income country. Our study showed that the improvement of data quality, characterized by the addition of one more identifier (mother date of birth), led to a significant improvement in the linkage quality, which increased the proportion of births for which you were able to identify a link in more recent years, reaching more than 80% proportion of highly probably link. Our comparison of missed-matches indicates a slightly higher proportion of missing data among missed matches and a higher proportion of people living in an urban area and self-declared as Caucasian among linked pairs when compared with non-linked sets.

An essential consideration of this linkage is the massive amount of data, which increases the technical complexity to perform the linkage process in a scalableway. The innovation of the CIDACS-RL is the use of the search engine indexing as a blocking strategy [18]. A traditional blocking strategy is applied to reduce the number of potential records comparisons that likely do not match and avoid waste of computational resources. However, this strategy can result in linkage error if true matches were separated in different blocks [20]. ‘To avoid linkage error without compromising the linkage scalability, CIDACS-RL implemented a dynamic search function that uses all linkage attributes for searching. This avoids computational waste similar to traditional blocking strategy without compromising the linkage quality, since it prevents linkage errors by non-separating in different blocks potential matches.

The use of a classical record linkage approach, as proposed by Fellegi and Sunter [21], was unfeasible. In this approach for each record pair, we calculated a probabilistic match weight based on two conditional probabilities: the probability of agreement given records belong to the same mother-baby pair (m-probability; P (agreement|match)), and the probability of agreement given records belong to different mother-baby pairs (u-probability, P (agreement|non-match)). However, we did not have these values, frequently provided by a gold-standard. Therefore our linkage cannot fit the ordinary probability-based classification model. The main difference between the CIDACS-RL method to the classical approach is the implementation of a similarity-based linkage that outputs the best pair of records and its similarity.

On the probabilistic linkage approach, the choice of thresholds is not straightforward, and it is going to impact directly on linkage quality. Decisions about the best thresholds are usually based on linkage scores of the complete dataset [11]. However, due to the massive amount of data, manual review for the complete dataset of comparison pairs was not possible. Therefore, it was selected a stratified sample size of 2000. The size of the sample was decided based on reasonability for manual revision that exhibited the same characteristics of the complete dataset on score distribution. The next step will be increasing the sample size and vary the characteristics of the sample and the linkage threshold to evaluate the linkage quality further.

Although linkage to enhance the same individual information can accomplish high sensitivity rates, the process of link information of two different people (in this case, mother and baby) has been considered a more problematic task, due to the limited number of shared identifiers within datasets [3, 22]. Which directly impacts on sensitivity results, which tend to be lower. In our study, the proportion of missed-matched records varied from 61% to 18%. In the first years of the study, our proportion of births for which you were able to identify a link based on a specified threshold was much lower than identified in similar studies in high-income countries. However, after the inclusion of the mother date of birth attribute, the proportion of missed-matches was similar to studies developed in the US States of Georgia [24] and New Jersey [23]. Another similarity with those studies was the higher proportion of vulnerable populations among residual records (rural, and worse living conditions).

This study has several limitations. A weakness of using the CIDACS birth cohort that should be addressed when answering individual research questions, is that it is restricted to people enrolled at CadUnico, which represents some of the poorest of Brazil's population. The main limitation inherent to the linkage process is the low proportion of births for which you were able to identify a link based on a specified threshold in the first years before the introduction of the mother's date of birth. This information is highly valuable because when using our cohort it could be decided to use only those years that have achieved the highest proportion of births for which you were able to identify a link. More important than the proportion of highly probably link of linkage in terms of proportion of links, the linkers have to guarantee that the linkage error did not introduce bias in the final analyses. Although the difference in some living conditions variables and socio-demographic’s characteristics between the linked and non-linked groups were less than 10%, even small amounts of linkage error can result in substantially biased results. For example, in the variable race, almost 25% of indigenous people were not linked; it could make a difference in studies using this population. Therefore, we recommend further studies to evaluate if these small differences can introduce bias and to take this in consideration in any future analyses using our birth cohort.

## Conclusion

An essential step of the linkage process is to estimate the linkage quality and to identify potential sources of bias that can be introduced in the results of analyses using the linked data. The linkage involving two nationwide large Brazilian databases evaluated here showed proportion of highly probably link for more recent years comparable with previous finds in developed countries [23, 24]. Although before the introduction of maternal date of birth in SINASC form , the proportion of missed match was much higher. The results presented in this study reinforce the need to evaluate linkage quality and to take linkage error into account as a preliminary step in the analyses of the linked datasets. However, the linkage of these datasets to form a large birth cohort is a valuable and much needed resource for future studies.

## Data Availability

The identified data used to conduct this study is highly sensible and confidential, because they include patient personal information that can be traced back to individual. They are obtainable in the Brazilian Ministry of Health but restrictions apply to the availability of these data, which were used under license, and so are not publicly accessible. However de-identified linked data can be accessed upon reasonable request for researchers who meet the criteria for access to confidential data.

## Abbreviations

SINASC: Live Birth Information System
CadUnico: Single Register for Social Programs
CIDACS: Centre for Data and Knowledge Integration for Health
ROC: Receiver operating curve
AUC: Area under the curves
